# Supplementary material for: Genome destabilization-associated phenotypes arising as a consequence of therapeutic treatment are suppressed by Olaparib
Source: PLoS One. 2023 Jan 27;18(1):e0281168. doi: 10.1371/journal.pone.0281168 (PMC9882903; doi:10.1371/journal.pone.0281168)
Supplement: S1 File — (PDF) [file pone.0281168.s001.pdf]

Supporting Information for

Genome Destabilization-Associated Phenotypes Arising as a Consequence of Therapeutic Treatment are Suppressed by Olaparib

Suzuki et al.

This PDF file includes:

S1 Table

S1 Fig.

S1 Table. Primers used for real time PCR.

b-actin-F1	CCTGGCACCCAGCACAAT
b-actin-R1	GCCGATCCACACGGAGTA
IFN-β-F1	TCTCCTCCAAATTGCTCTCC
IFN-β-R1	CTCCCATTCAATTGCCACAG
IFIT1-F1	GGAATACACAACCTACTAGCC
IFIT1-R1	CCAGGTCACCAGACTCCTCA
IFIT3-F1	TGAGGAAGGGTGGACACAACTGAA
IFIT3-R1	AGGAGAATTCTGGGTTGTTGGGCT

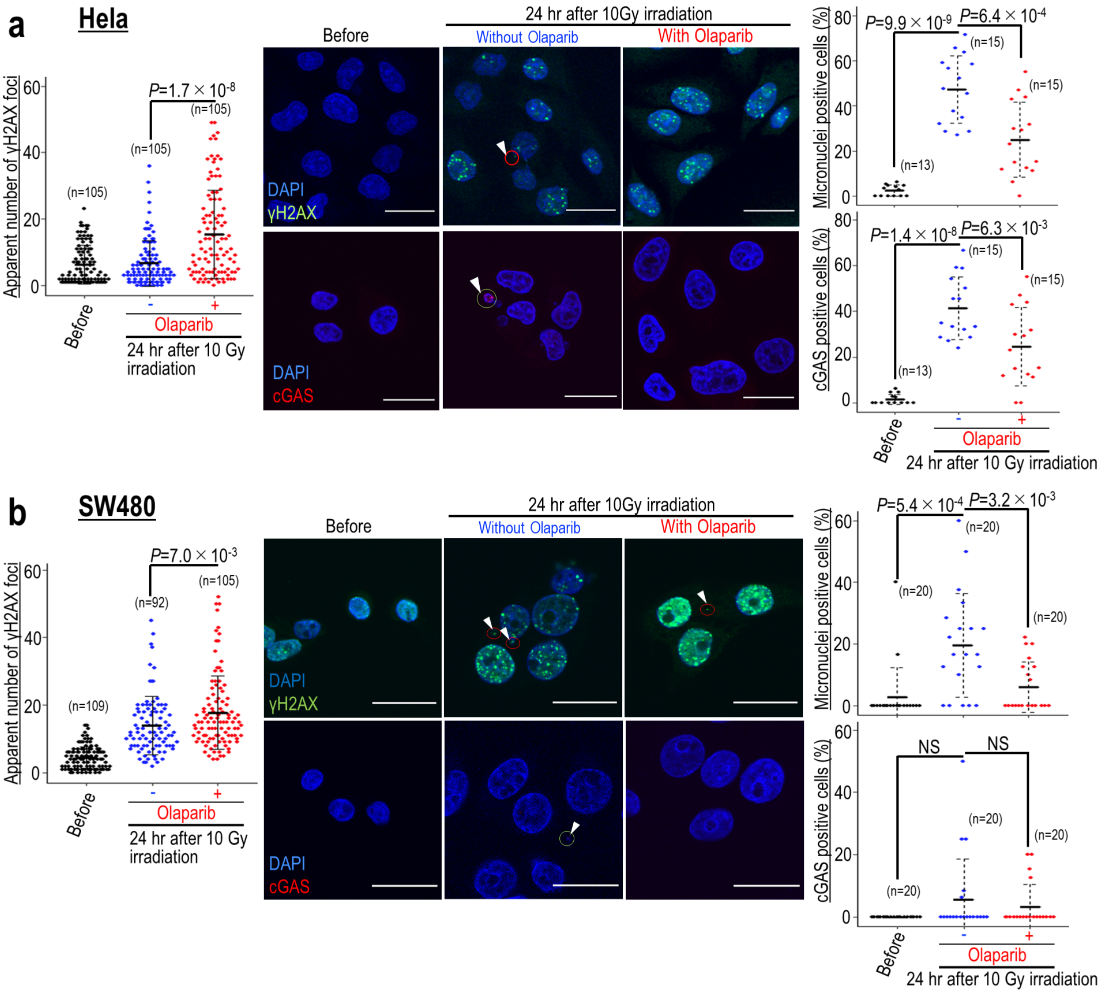

S1 Fig. Sensitized DSB responses in HeLa and SW480 cells in the presence of Olaparib. (A,B) HeLa cells **(A)** and SW480 cells **(B)** were irradiated with 10 Gy γ-ray in the presence and the absence of Olaparib, and cultivated for 24 hr. Numbers of γH2AX foci (Left panels) were plotted together and separately in the absence (blue dots) and the presence (red dots) of Olaparib. Micronuclei were identified by immunofluorescence staining for γH2AX and DAPI (Right top panels). Arrowheads show cGAS positive micronuclei foci (Right bottom panels). Representative images are provided. Scale bars in images, 30 µm. Bars show means ± s.d. Two-tailed Welch's t-test was used for statistical analysis. NS, not significant.
